# The performance of duplex ultrasonography for the assessment of renal artery stenosis in Takayasu’s arteritis patients

**DOI:** 10.1186/s13075-023-03121-8

**Published:** 2023-08-03

**Authors:** Yahong Wang, Ying Wang, Li Zhang, Zhitong Ge, Jing Li, Yunjiao Yang, Yu Chen, Xiao Yang, Jianchu Li, Xinping Tian

**Affiliations:** 1 Department of Ultrasound, State Key Laboratory of Complex Severe and Rare Diseases, Peking Union Medical College Hospital, Chinese Academy of Medical Sciences & Peking Union Medical College, Beijing, 100730 China; 2 Department of Rheumatology and Clinical Immunology, State Key Laboratory of Complex Severe and Rare Diseases, Peking Union Medical College Hospital, Chinese Academy of Medical Sciences & Peking Union Medical College, Beijing, 100730 China; 3 Department of Radiology, State Key Laboratory of Complex Severe and Rare Diseases,, Peking Union Medical College Hospital, Chinese Academy of Medical Sciences & Peking Union Medical College, Beijing, 100730 China

**Keywords:** Takayasu’s arteritis, Ultrasonography, Renal artery stenosis

## Abstract

**Background:**

This study intends to analyze the hemodynamic parameters of the renal artery in patients with Takayasu’s arteritis (TAK) to explore the diagnostic efficacy of duplex ultrasonography in assessing the involved renal artery in TAK patients.

**Methods:**

One hundred fifteen TAK patients with 314 renal arteries were retrospectively analyzed, who were admitted to Peking Union Medical College Hospital between 2017 and 2022. These patients underwent both renal artery ultrasonography and angiography within a 4-week period. Specifically, the study compared seven ultrasonic parameters across groups categorized by the severity of renal artery stenosis (RAS), including noninvolvement, < 50% stenosis, 50–69% stenosis, and 70–99% stenosis. Receiver operating characteristic (ROC) curves were employed to determine the optimal threshold values for renal artery peak systolic velocity (RPSV), renal-aortic PSV ratio (RAR), and renal-interlobar PSV ratio (RIR) in order to diagnose various degrees of RAS in TAK patients.

**Results:**

Statistically significant differences were observed in RAR and RIR among the four groups (all *P* < 0.05). However, no statistically significant differences were found in RPSV and AT between the moderate stenosis group (50–69% stenosis) and the severe stenosis group (70–99% stenosis). The discrimination of interlobar PSV (IPSV) and interlobar RI (IRI) was not significant, and IEDV did not show statistical significance among the four groups.

For TAK patients, the recommended thresholds of RPSV for the diagnosis of renal artery involvement (RAI), ≥ 50% RAS, and ≥ 70% RAS were determined to be 143 cm/s, 152 cm/s, and 183 cm/s, respectively. The sensitivities, specificities, and accuracies of these thresholds were all found to be greater than 80%. Additionally, the optimal thresholds of RIR for detecting RAI, ≥ 50% RAS, and ≥ 70% RAS were determined to be 4.6, 5.6, and 6.4, respectively, with satisfactory diagnostic efficiencies. The areas under the curve (AUCs) for RPSV and RIR were calculated to be 0.908 and 0.910, respectively, for the diagnosis of ≥ 50% RAS, and 0.876 and 0.882 for the diagnosis of ≥ 70% RAS. When the aortic PSV is greater than or equal to 140 cm/s, the RAR exhibits inadequate diagnostic efficacy. Conversely, when the aortic PSV is less than 140 cm/s, a RAR value of 2.2 or higher can be employed as the diagnostic threshold for identifying RAS of 70% or greater, with a sensitivity of 84.00%, specificity of 89.93%, and an overall accuracy of 89.08%.

**Conclusion:**

In the present study, it has been demonstrated that RPSV and RIR possess substantial diagnostic value as ultrasonic parameters for diagnosing RAS in TAK patients. Furthermore, when assessing the diagnostic efficacy of RAR, it is crucial to consider the severity of aortic stenosis as a determining factor.

## Background

Takayasu’s arteritis (TAK) is a rare chronic systemic vasculitis that mainly affects the aorta and its major branches [1]. The renal artery is one of the commonly involved vessels in TAK. It is reported that almost half of Asian TAK patients exhibit renal artery involvement [2, 3], resulting in the development of renal artery stenosis (RAS) or even occlusion, leading to hypertension or ischemic kidney injury [4, 5].

Duplex ultrasonography is a conventional imaging technique commonly employed for assessing the renal artery, yielding favorable diagnostic outcomes for RAS [6, 7]. However, the existing ultrasound diagnostic criteria for RAS are predominantly derived from studies involving patients with atherosclerotic RAS. TAK often leads to multiple vascular stenosis or abnormal cardiac structure and function, thereby inducing alterations in the comprehensive hemodynamics of the cardiovascular system. Therefore, previous ultrasound diagnostic criteria may not be applicable to TAK-involving renal arteries, but no previous research has been conducted on this topic. The purpose of this study is to evaluate the diagnostic value of duplex ultrasonography in the involved renal artery of TAK patients by analyzing renal artery hemodynamic parameters.

## Methods

### Patients

This study was based on an ultrasonic monitoring database of the Takayasu arteritis cohort, which was established in August 2017 at Peking Union Medical College Hospital (PUMCH), a Chinese national referral center. We retrospectively reviewed 559 medical charts of TAK patients until December 2022. Specifically, patients who had gone through both renal artery ultrasonography and either aortic computed tomography angiography (CTA) or digital subtraction angiography (DSA) within a 4-week period were included in the analysis. If a patient underwent multiple examinations that satisfied the inclusion criteria, only the latest examination data were included in the study. Patients who underwent renal artery stenting were excluded from the analysis. Ultimately, a total of 151 patients with technically satisfactory ultrasonic images and complete datasets were included. Among these patients, 8 cases had a unilateral accessory renal artery and 2 cases had a bilateral accessory renal artery, resulting in a total of 314 renal arteries being analyzed.

The diagnosis fulfills both the 1990 American College of Rheumatology (ACR) criteria and the 2022 ACR/European Alliance of Associations for Rheumatology (EULAR) classification criteria for TAK [8, 9]. The angiographic involvement pattern was assessed according to the criteria proposed by Numano et al. in 1994 [10], and the description records of angiography were analyzed. The thickening of the renal artery wall is considered renal artery involvement (RAI), while a reduction of ≥ 50% in diameter is considered to signify significant RAS.

### Ultrasound assessment

All patients were scanned by 4 experienced physicians who were proficient in vascular sonographic examination and interpretation with 10 to 15 years of experience. These four sonographers received training in standardized vascular ultrasound examination and were designated to conduct such examinations on patients in the TA cohort. A Philips system (IU 22 instrumentation) was used, with low-frequency 1- to 5-MHz curved linear phase array transducers. If the patient is thinner, a 3- to 9-MHz linear array probe was used to observe the initial segment of the bilateral renal arteries in the supine position. The angle of insonation was set at 60° or less, and the smallest possible Doppler angle was achieved by adjusting scanning sections to obtain a more substantial peak systolic velocity (PSV). Patients were examined in the anterior and lateral decubitus positions after an overnight fast to ensure a comprehensive examination of all sections of the main renal artery, from its origin to the hilum. In the left 45° recumbent position, using the descending liver as the sound transmission window during deep inspiration, the whole course of the right renal artery can be shown on the transverse section of the renal hilum, which is the preferred method for the observation of the right renal artery.

The PSV in the abdominal aorta was recorded at a distance of 1 cm below the point of the origin of the superior mesenteric artery. The highest renal artery PSV (RPSV) acquired at the narrowed site was recorded and selected to calculate the renal-aortic PSV ratio (RAR) and renal-interlobar PSV ratio (RIR). If there is no obvious narrow segment, the PSV of the proximal segment of the renal artery is selected. The spectrum of the interlobar renal artery was measured in the lateral position. Doppler spectra were elicited in the upper-, middle-, and lower-pole interlobar renal arteries along the pyramids, and the one with the most marked slope was selected for recording the interlobar PSV, EDV, acceleration time (AT), and RI. If no notable difference was found in the waveforms of early systole among these 3 sites, the middle-pole interlobar renal arteries were selected to record these Doppler parameters. To minimize errors in the measurement of these Doppler parameters, the following measures were adopted: (1) the smallest velocity scale and lowest wall filter setting were used; (2) a medium sweep speed was set; and (3) parameters were recorded from 1 of at least 3 of the same waveforms obtained when the patient held breath.

### Statistical analysis

Statistical analyses were conducted by SPSS software (version 27.0). Numerical data was expressed as mean ± standard deviation (SD) or median (range: minimum to maximum), while categorical data was expressed as percentages or numbers. Numerical data were compared using the independent sample* t*-test or the one-way analysis of variance, followed by the least significant difference comparison method. Categorical data were compared using the chi-square or Fisher exact test, as appropriate. All probabilities were two-sided, and *P* values < 0.05 were considered statistically significant.

The diagnostic efficacy of renal artery hemodynamic parameters (RPSV, RAR, and RIR) in the evaluation of RAS was analyzed by using angiography as the diagnostic standard. Receiver operating characteristic (ROC) curves and Youden’s index were used to calculate optimal cutoff values for RAS.

## Results

Among the 151 patients, 142 were female and 9 were male, aged 4–73 years, with a median age of 29 (23, 34) years. Eighty-one individuals (53.6%) of them exhibited unilateral or bilateral renal artery involvement. Table 1 shows the angiographic manifestations of the 151 patients. The most prevalent pattern of angiographic involvement, observed in 78.1% of cases, was Type V, characterized by extensive involvement of the aorta and its main branches. The proportion of significant aortic stenosis (≥ 50%) above the origin of the renal artery was 15.3%. However, other 46.4% of the aorta exhibited mild stenosis (30–49% stenosis), which also might affect the blood flow of the renal artery. One hundred thirty-one (41.7%) of the 314 renal arteries were involved, and most of them affect the proximal segment (81.7%). Echocardiography revealed mild aortic valve regurgitation in 43 (28.4%) patients and moderate to severe aortic valve regurgitation in 13 (8.6%) patients.

**Table 1:** Angiographic findings in the 151 TAK patients (314 renal arteries)

Variable	No. (%)
**Angiographic classification**	
Type I	9 (6.0%)
Type IIa	4 (2.6%)
Type IIb	13 (8.6%)
Type III	4 (4.6%)
Type IV	3 (2.0%)
Type V	118 (78.1%)
**Degree of aortic stenosis above the level of renal artery origin**	
< 30%	58 (38.4%)
30–49%	70 (46.4%)
50–69%	17 (11.3%)
70–99%	6 (4.0%)
**Renal artery involvement**	131 (100%)
**Left/right**	
Right	59 (45.0%)
Left	72 (55.0%)
** Involved segment**	
Proximal	107 (81.7%)
Middle/distal	2 (1.5%)
All segments	22 (16.8%)
**Degree of stenosis**	
< 50%	41 (31.3%)
50–69%	24 (18.3%)
70–99%	50 (38.2%)
Occlusion	16 (12.2%)
Dilatation/aneurysm	0

Sixteen occluded renal arteries, one whose interlobar artery spectrum could not be measured due to renal atrophy, and six with unsatisfactory imaging at the stenosis due to excessive bowel gas were excluded. The remaining 291 renal arteries were included in the statistical analysis.

Table 2 shows the differences in hemodynamic parameters significant stenosis group (≥ 50% stenosis) and non-significant stenosis group (< 50% stenosis). It is worth mentioning that all parameters, except for interlobar renal artery EDV (IEDV), exhibited statistically significant difference between the two groups (all *P* < 0.001). Furthermore, Table 3 illustrates the variations in hemodynamic parameters across different degrees (noninvolvement, < 50% stenosis, 50–69% stenosis, 70–99% stenosis) of RAS. The results showed that there were statistically significant variations in RAR and RIR across the four groups (all *P* < 0.05). However, no statistically significant differences were observed in RPSV and AT between the moderate stenosis group (50–69% stenosis) and the severe stenosis group (70–99% stenosis). The discriminatory ability of interlobar PSV (IPSV) and interlobar RI (IRI) was not found to be significant, while IEDV did not exhibit statistical significance among the four groups.

**Table 2:** Comparison of hemodynamic parameters between significant stenosis group and non-significant stenosis group of RAS in TAK patients

Parameter		< 50% stenosis	≥ 50% stenosis	Value	*P*
		(*n* = 222)	(*n* = 69)		
RPSV (cm/s)		112.50 (90.00,139.00)	256.00 (190.00,372.50)	− 10.341	< 0.001
RAR		0.92 (0.67,1.23)	1.50 (1.21,2.90)	− 7.997	< 0.001
RIR		3.20 (2.40,4.36)	8.12 (6.31,15.73)	− 10.477	< 0.001
IPSV (cm/s)		36.67 ± 12.13	30.51 ± 14.04	3.541	< 0.001
IEDV (cm/s)		11.82 ± 4.47	12.29 ± 4.95	− 0.751	0.454
IRI		0.66 ± 0.11	0.58 ± 0.14	5.398	< 0.001
AT	< 0.07 s	180 (81.1%)	28 (40.6%)	42.354	< 0.001
	≥ 0.07 s	42 (18.9%)	41 (59.4%)

**Table 3:** Comparison of hemodynamic parameters among groups with different degrees of RAS in TAK patients

Parameter		Noninvolvement	< 50% stenosis	50–69% stenosis	70–99% stenosis	Value	*P*
		(*n* = 182)	(*n* = 40)	(*n* = 23)	(*n* = 46)		
AT	< 0.07 s	152 (83.5%)	28 (70.0%)	9 (39.1%)	19 (41.3%)	45.328	< 0.001
	≥ 0.07 s	30 (16.5%)a	12 (30.0%)b	14 (60.9%)c	27 (58.7%)c		
RPSV (cm/s)		106.00 (87.00, 130.00)a	174.00 (116.00, 227.00)b	219.00 (185.25, 307.50)c	300.00 (195.00, 404.00)c	134.040	< 0.001
IPSV (cm/s)		36.05 ± 11.43ab	39.40 ± 14.77a	31.00 ± 8.31bc	30.26 ± 16.25c	4.973	0.002
IEDV (cm/s)		11.85 ± 4.39	11.65 ± 4.88	11.65 ± 3.83	12.61 ± 5.43	0.429	0.732
IRI		0.66 ± 0.11ab	0.69 ± 0.11a	0.62 ± 0.10bc	0.57 ± 0.15d	12.450	< 0.001
RAR		0.89 (0.66, 1.16)a	1.11 (0.76, 1.38)b	1.41 (1.07, 1.98)c	2.25 (1.34, 3.45)d	72.282	< 0.001
RIR		3.11 (2.33, 3.95)a	4.72 (2.90, 6.65)b	6.90 (5.92, 9.24)c	10.00 (6.42, 16.65)d	123.597	< 0.001

The same letter indicates that there was no statistically significant difference between the groups (*P* > 0.05), and the different letters indicate that the difference between the groups was statistically significant (*P* < 0.05)

**Fig. 1:**
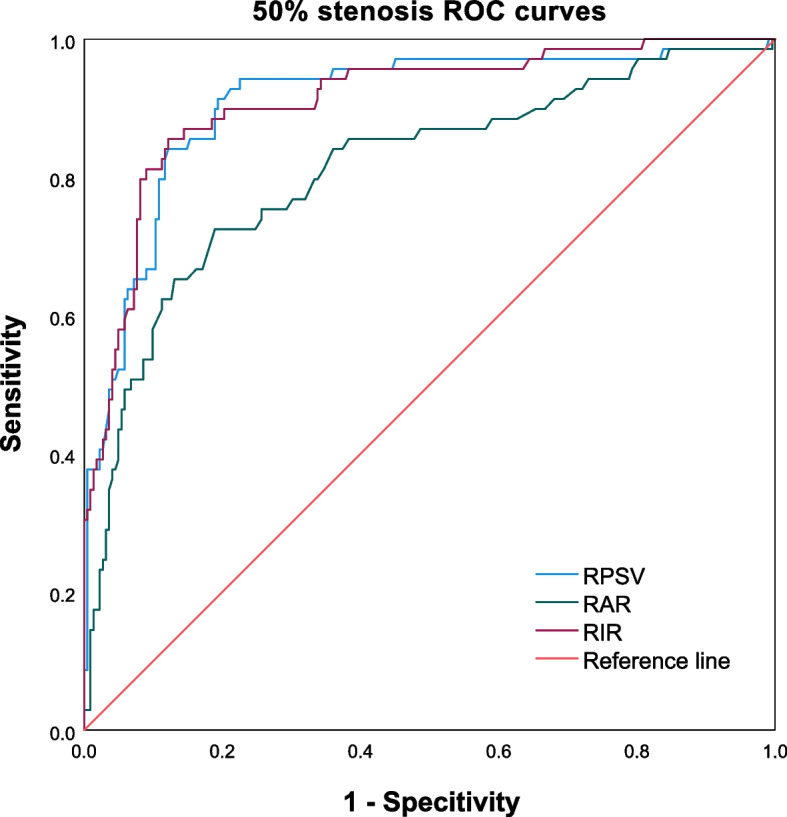
Receiver operating characteristic (ROC) curves are shown for ≥ 50% RAS in TAK patients

**Fig. 2:**
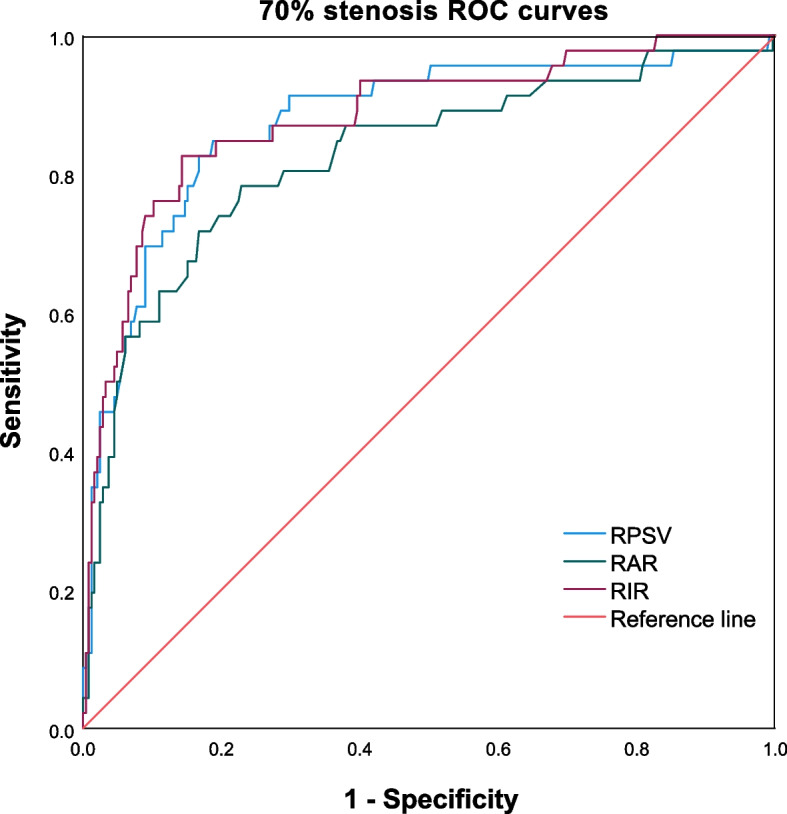
Receiver operating characteristic (ROC) curves are shown for ≥ 70% RAS in TAK patients

Table 4 summarizes the cutoff values and diagnostic efficacies of RPSV, RAR, and RIR in detecting different degrees of RAS in TAK patients. The ROC curves for ≥ 50% and ≥ 70% RAS in TAK patients are depicted in Figs. 1 and 2, respectively. It is worth noting that the cutoff values for RPSV in detecting renal artery involvement (RAI), ≥ 50% RAS, and ≥ 70% RAS are 143 cm/s, 152 cm/s, and 183 cm/s, respectively. Meanwhile, the sensitivity, specificity, positive predictive value (PPV), negative predictive value (NPV), and accuracy of these measurements exceed 80%. The cutoff values for RIR in detecting RAI, ≥ 50% RAS, and ≥ 70% RAS are 4.6, 5.6, and 6.4, respectively. However, the cutoff values for RAR were indistinct, with values of 1.1,1.3, and 1.3, respectively. Additionally, the diagnostic performance of RAR is inferior to that of RPSV and RIR. The area under the curve (AUC) for RAR, RPSV, and RIR are 0.811, 0.908, and 0.910, respectively, for ≥ 50% RAS, and 0.825, 0.876, and 0.882, respectively, for ≥ 70% RAS in TAK patients.

**Table 4:** The cutoff values and diagnostic efficacies of hemodynamic parameters for detecting different degrees of RAS in TAK patients

Parameter		Cutoff value	AUC	Sensitivity (%)	Specificity (%)	PPV (%)	NPV (%)	Accuracy (%)
RPSV	Involvement	143	0.882	83.49	86.81	79.13	89.78	85.57
	≥ 50% RAS	152	0.908	91.30	80.63	59.43	96.76	83.16
	≥ 70% RAS	183	0.876	84.78	81.22	41.77	97.11	81.79
RAR	Involvement	1.1	0.754	74.31	68.68	58.69	81.70	70.79
	≥ 50% RAS	1.3	0.811	72.46	81.08	54.35	90.45	79.04
	≥ 70% RAS	1.3	0.825	78.26	77.14	39.13	94.97	77.32
RIR	Involvement	4.6	0.847	76.15	86.81	77.57	85.87	82.82
	≥ 50% RAS	5.6	0.910	85.51	87.84	68.61	95.12	87.29
	≥ 70% RAS	6.4	0.882	82.61	85.71	52.05	96.33	85.22

*AUC* area under the curve, *NPV* negative predictive value, *PPV* positive predictive value

Given that 61.7% of the TAK patients exhibited mild or more severe aortic stenosis (≥ 30% stenosis) above the level of renal artery origin, the 291 renal arteries were categorized into two distinct groups based on whether their aortic PSV exceeded or fell below the threshold of 140 cm/s (the cutoff value for ≥ 30% aortic stenosis). Table 5 shows the cutoff values and diagnostic efficacies of RAR in detecting different degrees of RAS in the two groups. It is observed that the cutoff value of RAR for detecting ≥ 70% RAS differs significantly from that for ≥ 50% RAS (2.2 versus 1.4), as well as from that for ≥ 70% RAS when not stratified by the aortic PSV (2.2 versus 1.3), when the aortic PSV is below 140 cm/s. However, when the aortic PSV is ≥ 140 cm/s, the differences in the cutoff values of RAR for detecting different degrees of RAS are less pronounced (0.9, 1.0, and 1.1, respectively).

**Table 5:** The cutoff values and diagnostic efficacies of RAR for detecting different degrees of RAS in aortic PSV < 140 cm/s group and aortic PSV ≥ 140 cm/s group

Aortic PSV	No		Cutoff value	AUC	Sensitivity (%)	Specificity (%)	PPV (%)	NPV (%)	Accuracy (%)
< 140 cm/s	174	Involvement	1.4	0.840	74.51	86.99	70.37	89.17	83.33
		≥ 50% RAS	1.4	0.888	88.24	82.86	55.56	96.67	83.91
		≥ 70% RAS	2.2	0.885	84.00	89.93	58.33	97.10	89.08
≥ 140 cm/s	117	Involvement	0.9	0.839	68.97	89.83	86.96	74.65	79.49
		≥ 50% RAS	1.0	0.848	77.14	84.15	67.51	89.61	82.05
		≥ 70% RAS	1.1	0.838	80.95	80.21	47.22	95.06	80.34

*AUC* area under the curve, *NPV* negative predictive value, *PPV* positive predictive value

## Discussion

Takayasu’s arteritis often involves young women, which can cause multiple vascular stenosis, leading to target organ function damage, including the heart, brain, kidneys, limbs, and even fertility in young women. The growing interest among rheumatologists in this disease, coupled with extensive research involving large sample sizes, has contributed to significant advancements in the diagnosis and treatment of Takayasu’s arteritis in recent years [9, 11, 12]. Consequently, this progress has also facilitated the development of imaging techniques for the diagnosis and evaluation of TAK.

The renal artery is a commonly involved vessel in TAK, although studies in different regions have shown that the proportion of renal artery involvement (RAI) varies. The prevalence of RAI among TAK patients varies from 11.5 to 62%, but approximately half of Asian and Mexican TAK patients have RAI [4, 13, 14]. Two extensive studies conducted on Chinese TAK patients reported RAI proportions of 30.34% and 48.90%, respectively [4, 5]. The proportion (53.6%) of patients with renal artery involvement was relatively high in our study, which was related to the selection bias of patients.

Unlike atherosclerosis, the TAK-involved renal artery wall is often thickened circumferentially with rare instances of calcification. TAK predominantly affects the proximal segment of the renal artery, mainly causing stenosis, with a smaller proportion of cases involving arterial dilatation or aneurysm. The present study observed a high prevalence of proximal segment involvement, accounting for 81.7% (107/131) of cases, followed by the involvement of the entire main trunk (16.8%). The distribution of TAK involvement was similar between the left and right sides, with 55.0% affecting the left side and 45.0% (right) affecting the right side. Chen et al. reported that only 5 to 8% of RAIs manifested as renal artery dilatation or aneurysm, and are always pre- or post-stenotic [4]. However, we did not find these changes in our study. In our sample, the proportion of severe stenosis and occlusion was found to be 50.4% in RAIs, whereas a previous study indicated that severe stenosis was one of the predictors for medium-term adverse outcomes [5].

TAK frequently affects the aorta and its branches widely, resulting in multiple vascular stenosis, of which type V is the most common type, followed by type I. Consequently, a higher proportion of aortic stenosis is observed in TAK compared to atherosclerosis, and suprarenal aortic stenosis can significantly impact ultrasonic hemodynamic parameters, including RPSV, IPSV, RI, and AT, among others. Secondly, due to the stenosis of multiple branches such as the brachiocephalic trunk, the redistribution of systemic blood flow may be caused, so the blood perfusion status of the renal artery in TAK patients may differ from those without TAK-related renal artery stenosis. Thirdly, as mentioned above, the renal artery stenosis in TAK patients primarily manifests as segmental and circumferential stenosis, which is different from the localized and eccentric stenosis typically caused by atherosclerotic plaque. Consequently, the hemodynamic status and ultrasonic parameters of TAK-related stenosis may differ from those observed in atherosclerosis. However, it is worth mentioning that the existing ultrasound diagnostic criteria for RAS, as established in previous studies, predominantly rely on data obtained from patients with atherosclerotic RAS, most of whom are elderly and have different disease characteristics. Therefore, the existing ultrasound diagnostic criteria may not be applicable to TAK-involving renal arteries, and it is necessary to conduct independent research on the ultrasound diagnostic indicators of renal artery stenosis in TAK patients.

According to the literature we have reviewed, this study represents the most extensive sample size analysis to date that systematically analyzes the ultrasound diagnostic parameters of RAS in TAK patients. There have been few studies on TAK-involving RAS, and all of them are included in the samples of RAS caused by various causes. AbuRahma et al. conducted the largest study in terms of sample size, but no TAK patients were included in the 313 patients [7]. On the other hand, Li et al. included the highest number of TAK patients, but there were only 29 TAK patients out of a total of 81 patients. Furthermore, their evaluation focused solely on the differences in the interlobar artery AT and RI between individuals with atherosclerotic and nonatherosclerotic RAS [15].

This study shows that both RPSV and RIR exhibit favorable diagnostic efficacies in identifying renal artery involvement and renal artery stenosis in TAK patients. The diagnostic accuracies are both more than 80%, and the cutoff values for different degrees of stenosis exhibit satisfactory discrimination, which is convenient for clinical application. A meta-analysis study showed that RPSV exhibits superior performance characteristics, with an expected sensitivity of 85% and specificity of 92% [16]. It should be noted that the diagnostic thresholds used in various research centers are different. The commonly used diagnostic thresholds of ≥ 50% or ≥ 60% RAS typically correspond to velocities of 180 cm/s or 200 cm/s, yielding sensitivities and specificities ranging from 85 to 90% [6, 17–22]. In the case of TAK patients, when the renal artery is involved, the PSV usually exceeds 143 cm/s, and when PSV is greater than 152 cm/s or 183 cm/s, it indicates that the degree of RAS is equal to or exceeds 50% or 70%, respectively, as demonstrated in this study.

RIR is the ratio of RPSV to IPSV, and it is less affected by factors such as aortic disease or cardiac function; thus, this index is one of the reliable indicators for ultrasound diagnosis of renal artery stenosis. Similar parameter is RSR, which is the ratio of RPSV to the PSV of the segmental artery. Souza de Oliveira et al. used ROC curve analysis to obtain that the optimal threshold of RSR for diagnosing ≥ 50% RAS was 5, yielding a sensitivity of 93.33% and specificity of 89.47% [23]. Similarly, Li et al. demonstrated that the optimal diagnostic threshold of RIR for ≥ 50% RAS was 5.5, with an AUC of 0.927, and both sensitivity and specificity were reported to be 88% [24]. For TAK patients, our study showed that the optimal thresholds for diagnosing RAI, ≥ 50% RAS, and ≥ 70% RAS were determined to be 4.6, 5.6, and 6.4, respectively. Notably, the diagnostic threshold of ≥ 50% RAS (5.6) closely aligned with the findings of Li et al. (5.5), indicating that the influence of etiology on RIR may be minimal.

For RAR, this study proposes that aortic stenosis occurring above the origin of the renal artery significantly affects the performance of it. Taking no account of aortic stenosis, the cutoff values and diagnostic accuracies of RAR were suboptimal, and it demonstrated limited ability to differentiate between RAI, ≥ 50% RAS, and ≥ 70% RAS in TAK patients. The respective cutoff values for RAI, ≥ 50% RAS, and ≥ 70% RAS were 1.1, 1.3, and 1.3, with corresponding diagnostic accuracies of 70.79%, 79.04%, and 77.32%, respectively. When categorized based on whether the aortic PSV at the level of renal artery opening was ≥ 140 cm/s (the optimal cutoff value for ≥ 30% aortic stenosis), the diagnostic effectiveness of RAR was significantly enhanced when the aortic PSV was < 140 cm/s, and meantime the diagnostic threshold for RAS ≥ 70% was determined to be 2.2, with a sensitivity of 84.00%, specificity of 89.93%, and diagnostic accuracy of 89.08%. But, when aortic stenosis was ≥ 30% or aortic PSV was ≥ 140 cm/s, the diagnostic effectiveness of RAR was not satisfactory. Therefore, for TAK patients, RAR should not be used as a diagnostic indicator when the abdominal aorta is narrow or the aortic PSV is increased (≥ 140 cm/s). Conversely, when the abdominal aorta is within normal limits or the stenosis is less than 30%, a diagnosis of RAS ≥ 70% can be established if RAR ≥ 2.2. This diagnostic threshold is comparatively lower than that of non-TAK RAS (2.5 ~ 3.7, mostly 3.5) [6, 7, 20, 25–27], indicating a decrease in the disparity between the PSV of the abdominal aorta and the PSV of the narrow renal artery in TAK patients, which may be attributed to the hemodynamic alterations occurring within the systemic circulation system of TAK patients.

For the hemodynamic parameters of the interlobar artery, IEDV had no significant difference among groups of different stenosis degrees, while the differences in IPSV, IRI, and AT among these groups were not as significant as those observed in RPSV and RIR. In addition, due to aorta or coronary artery involvement, some patients might also have cardiac structural and functional abnormalities, such as aortic valve insufficiency. In this group of TAK patients, the proportion of aortic valve insufficiency was as high as 37.0%, with 8.6% of cases classified as moderate to severe. Aortic valve insufficiency can lead to a decrease in diastolic blood flow and velocity within the renal artery, which directly leads to an increase in renal artery RI. Therefore, in TAK patients, the increase of renal artery RI is not necessarily caused by renal impairment but may also be caused by cardiac problems such as aortic valve insufficiency, which needs comprehensive analysis.

The major limitation of this study is that most of the TAK patients underwent CTA examination rather than DSA, because the use of catheter-based arteriograms has been largely replaced by noninvasive imaging modalities in TAK patients nowadays [28]. Therefore, there may be the possibility of overestimating or underestimating the degree of RAS in some cases, which may compromise the power of the conclusion. However, the diagnostic accuracy of CTA for detecting renal artery stenosis has been reported to be as high as 97.8% [29, 30]. Consequently, CTA has become a widely utilized method for evaluating arterial involvement in TAK patients. In the case of a large sample size, it can reduce the impact on the research conclusions. In addition, the proportion of TAK patients who underwent renal artery ultrasonography and angiography within a 4-week period was relatively small, and further prospective study is needed.

## Conclusions

In conclusion, our study has indicated that RPSV and RIR remain the most valuable ultrasonic diagnostic parameters for assessing RAS, even in TAK patients, but the optimal thresholds for these parameters seem to differ from the existing diagnostic criteria established based on atherosclerotic patients. For TAK patients, our data suggests the optimal thresholds of RPSV for diagnosing ≥ 50% RAS and ≥ 70% RAS were determined to be 152 cm/s and 183 cm/s, respectively; meanwhile, the optimal thresholds of RIR were identified as 5.6 and 6.4, respectively. For RAR, its diagnostic value can be judged according to the severity of aortic stenosis. In our study, when the aortic PSV is ≥ 140 cm/s, RAR is not considered a satisfied parameter for diagnosing RAS. However, when the aortic PSV is < 140 cm/s, a RAR value ≥ 2.2 can serve as the diagnostic threshold for identifying renal artery stenosis of ≥ 70%.

Additional research is warranted to explore the potential influence of cardiac-related issues on the hemodynamics of patients diagnosed with Takayasu’s arteritis.

## Data Availability

The datasets used and/or analyzed during the current study are available from the corresponding author on reasonable request.

## Abbreviations

AT: Acceleration time
CTA: Computed tomography angiography
DSA: Digital subtraction angiography
EDV: End-diastolic velocity
IPSV: PSV of interlobar renal artery
PSV: Peak systolic velocity
RAI: Renal artery involvement
RAR: Renal-aortic PSV ratio
RPSV: PSV of renal artery at the narrow site
RI: Resistant index
RIR: Renal-interlobar PSV ratio
TAK: Takayasu’s arteritis

## References

[1] Jain S, Pondaiah SK (2015). Takayasu’s arteritis: review of epidemiology and etiopathogenesis. Indian J Rheumatol.

[2] Alibaz-Oner F, Direskeneli H (2015). Update on Takayasu’s arteritis. Presse Med.

[3] Li J, Sun F, Chen Z (2017). The clinical characteristics of Chinese Takayasu's arteritis patients: a retrospective study of 411 patients over 24 years. Arthritis Res Ther.

[4] Chen Z, Li J, Yang Y (2018). The renal artery is involved in Chinese Takayasu's arteritis patients. Kidney Int.

[5] Sun Y, Dai X, Lv P (2021). Characteristics and Medium-term Outcomes of Takayasu Arteritis-related Renal Artery Stenosis: Analysis of a Large Chinese Cohort. J Rheumatol.

[6] Zachrisson K, Herlitz H, Lönn L, Falkenberg M, Eklöf H (2017). Duplex ultrasound for identifying renal artery stenosis: direct criteria re-evaluated. Acta Radiol.

[7] AbuRahma AF, Srivastava M, Mousa AY (2012). Critical analysis of renal duplex ultrasound parameters in detecting significant renal artery stenosis. J Vasc Surg.

[8] Arend WP, Michel BA, Bloch DA (1990). The American College of Rheumatology 1990 criteria for the classification of Takayasu arteritis. Arthritis Rheum.

[9] Grayson PC, Ponte C, Suppiah R (2022). 2022 American College of Rheumatology/EULAR Classification Criteria for Takayasu Arteritis. Arthritis Rheumatol.

[10] Hata A, Noda M, Moriwaki R (1996). Angiographic findings of Takayasu arteritis: new classification. Int J Cardiol.

[11] Hellmich B, Agueda A, Monti S (2020). 2018 Update of the EULAR recommendations for the management of large vessel vasculitis. Ann Rheum Dis.

[12] Maz M, Chung SA, Abril A (2021). 2021 American College of Rheumatology/Vasculitis Foundation Guideline for the Management of Giant Cell Arteritis and Takayasu Arteritis. Arthritis Rheumatol.

[13] Lee GY, Jang SY, Ko SM (2012). Cardiovascular manifestations of Takayasu arteritis and their relationship to the disease activity: analysis of 204 Korean patients at a single center. Int J Cardiol.

[14] Khor CG, Tan BE, Kan SL (2016). Takayasu arteritis in major rheumatology centers in Malaysia. J Clin Rheumatol.

[15] Li JC, Yuan Y, Qin W (2007). Evaluation of the tardus-parvus pattern in patients with atherosclerotic and nonatherosclerotic renal artery stenosis. J Ultrasound Med.

[16] Williams GJ, Macaskill P, Chan SF (2007). Comparative accuracy of renal duplex sonographic parameters in the diagnosis of renal artery stenosis: paired and unpaired analysis. AJR Am J Roentgenol.

[17] Schäberle W, Leyerer L, Schierling W, Pfister K (2016). Ultrasound diagnostics of renal artery stenosis: Stenosis criteria CEUS and recurrent in-stent stenosis. Gefasschirurgie.

[18] Olin JW, Piedmonte MR, Young JR, DeAnna S, Grubb M, Childs MB (1995). The utility of duplex ultrasound scanning of the renal arteries for diagnosing significant renal artery stenosis. Ann Intern Med.

[19] Hoffmann U, Edwards JM, Carter S (1991). Role of duplex scanning for the detection of atherosclerotic renal artery disease. Kidney Int.

[20] Zeller T, Bonvini RF, Sixt S (2008). Color-coded duplex ultrasound for diagnosis of renal artery stenosis and as follow-up examination after revascularization. Catheter Cardiovasc Interv.

[21] Li JC, Jiang YX, Zhang SY, Wang L, Ouyang YS, Qi ZH (2008). Evaluation of renal artery stenosis with hemodynamic parameters of Doppler sonography. J Vasc Surg.

[22] Conkbayir I, Yücesoy C, Edgüer T, Yanik B, Yaşar Ayaz U, Hekimoğlu B. Doppler sonography in renal artery stenosis. An evaluation of intrarenal and extrarenal imaging parameters. Clin Imaging. 2003;27(4):256–260. 10.1016/s0899-7071(02)00547-8 12823921

[23] Souza de Oliveira IR, Widman A, Molnar LJ, Fukushima JT, Praxedes JN, Cerri GG. Colour Doppler ultrasound: a new index improves the diagnosis of renal artery stenosis. Ultrasound Med Biol. 2000;26(1):41–47. 10.1016/s0301-5629(99)00119-2 10687791

[24] Li JC, Wang L, Jiang YX (2006). Evaluation of renal artery stenosis with velocity parameters of Doppler sonography. J Ultrasound Med.

[25] Staub D, Canevascini R, Huegli RW (2007). Best duplex-sonographic criteria for the assessment of renal artery stenosis–correlation with intra- arterial pressure gradient. Ultraschall Med.

[26] Drelich-Zbroja A, Kuczyńska M, Światłowski Ł, Szymańska A, Elwertowski M, Marianowska A (2018). Recommendations for ultrasonographic assessment of renal arteries. J Ultrason.

[27] Chain S, Luciardi H, Feldman G (2006). Diagnostic role of new Doppler index in assessment of renal artery stenosis. Cardiovasc Ultrasound.

[28] Dua AB, Kalot MA, Husainat NM (2021). Takayasu Arteritis: a Systematic Review and Meta-Analysis of Test Accuracy and Benefits and Harms of Common Treatments. ACR Open Rheumatol.

[29] Fraioli F, Catalano C, Bertoletti L (2006). Multidetector-row CT angiography of renal artery stenosis in 50 consecutive patients: prospective interobserver comparison with DSA. Radiol Med.

[30] Zhang HL, Sos TA, Winchester PA, Gao J, Prince MR (2009). Renal artery stenosis: imaging options, pitfalls, and concerns. Prog Cardiovasc Dis.

